# NT-proBNP, Echocardiography Patterns and Outcomes in Sepsis-Induced Cardiac Dysfunction

**DOI:** 10.3390/jcm14248714

**Published:** 2025-12-09

**Authors:** Catalina Paraschiv, Denisa Oana Nicolaescu, Roxana Mihaela Popescu, Laura Barbalata, Emanuel Moisa, Silvius Negoita, Andreea Catarina Popescu, Serban Mihai Balanescu

**Affiliations:** 1 Department of Cardiology, Elias Emergency University Hospital, University of Medicine and Pharmacy “Carol Davila”, 050474 Bucharest, Romaniaserban.balanescu@umfcd.ro (S.M.B.); 2 Department of Cardiovascular Diseases, Mayo Clinic, Rochester, MN 55902, USA; 3 Department of Anesthesia and Intensive Care Medicine, Elias Emergency University Hospital, 011461 Bucharest, Romania

**Keywords:** sepsis, septic shock, systolic dysfunction, diastolic dysfunction, echocardiography, NT-proBNP

## Abstract

**Introduction:** Sepsis-induced cardiac dysfunction (SICD) is a reversible type of cardiac disorder, with variable reported incidence and uncertain prognostic implications that may occur in patients with sepsis. This study aimed to identify patients with SICD, characterise their clinical and paraclinical features, analyse the different patterns of cardiac dysfunction, and determine prognostic implications. **Methods:** Patients admitted to the intensive care unit (ICU) for sepsis or septic shock who underwent echocardiography on admission were identified retrospectively. Exclusion criteria included acute myocardial infarction, preexisting severe left heart valve disease or systolic dysfunction. Clinical, paraclinical, and echocardiography data were documented. The primary outcome was a composite of in-hospital mortality, prolonged hospital stay, and prolonged ICU stay. **Results:** A total of 128 septic patients were included, with a median age of 72.5 years and a 55% male proportion. Alcohol abuse, vasopressor therapy and orotracheal intubation on admission were risk factors for developing SICD. More than a third developed SICD (37%). We identified four different types of cardiac dysfunction based on echocardiography. An NT-proBNP level of over 9000 pg/mL was a predictor of SICD, composite outcome and mortality. A total of 56 patients (44%) experienced in-hospital mortality. Even though the occurrence of SICD did not predict mortality, it was a significant predictor of the composite outcome. **Conclusions:** This study describes the incidence and spectrum of SICD in a group of septic patients admitted to a tertiary care hospital. The occurrence of any type of cardiac dysfunction associated with sepsis and high NT-proBNP levels had strong prognostic implications.

## 1. Introduction

Sepsis is defined as a severe, potentially fatal organ dysfunction caused by an abnormal response to bacterial, viral, or parasitic factors [1]. However, in clinical practice, sepsis usually refers to a condition caused by bacterial infection. Despite recent advancements in antimicrobial treatments and critical care, sepsis remains a complex condition, with significant morbidity and mortality worldwide. Sepsis can lead to various complications and progressive organ failure, including cardiac dysfunction [1].

Sepsis-induced cardiac dysfunction (SICD) is a reversible type of cardiac disorder, with variable reported incidence and uncertain prognostic implications that can occur in patients with sepsis [2,3]. Although there is no universal definition for SICD, it is often defined as a reversible reduction in left ventricular ejection fraction (LVEF) of lower than 50% and a decrease of at least 10% compared to the baseline LVEF [3]. However, this characterization is incomplete. Right ventricular systolic dysfunction and left ventricular diastolic dysfunction associated with sepsis have also been described [4]. The occurrence of left ventricular (LV) systolic dysfunction in septic patients has been reported at around 20% [5]. An important criterion in SICD is the reversible nature of the dysfunction. The recovery time is typically 7–10 days, which would only apply to critically ill patients who survive that long. Several risk factors for developing SICD were identified in previous studies, including younger age, diabetes mellitus, a history of heart failure, higher lactate levels upon admission, polymicrobial cultures [6,7,8,9,10]. Previous reports mention higher inflammation markers and cardiac biomarkers levels in septic patients who develop SICD [7,10,11].

The pathophysiological mechanisms of SICD are still incompletely understood, with ongoing research targeting the interactions among several incriminated factors, such as inflammation mediators, calcium dysregulation, and mitochondrial dysfunction, all of which lead to myocardial cell injury [12]. The epidemiology, clinical, and paraclinical risk factors for developing this condition have not been fully described, highlighting the need for further studies to enhance our understanding and improve the diagnosis and management of SICD [13].

The primary aim of this study was to identify the incidence, the echocardiographic patterns, and prognostic implications of cardiac dysfunction in a group of patients admitted with sepsis. The secondary objectives were to identify the clinical and paraclinical risk factors determined on hospital admission that could predict the risk of developing cardiac dysfunction in septic patients. Understanding these aspects could facilitate early diagnosis and better patient management.

## 2. Materials and Methods

### 2.1. Study Design

**Figure 1:**
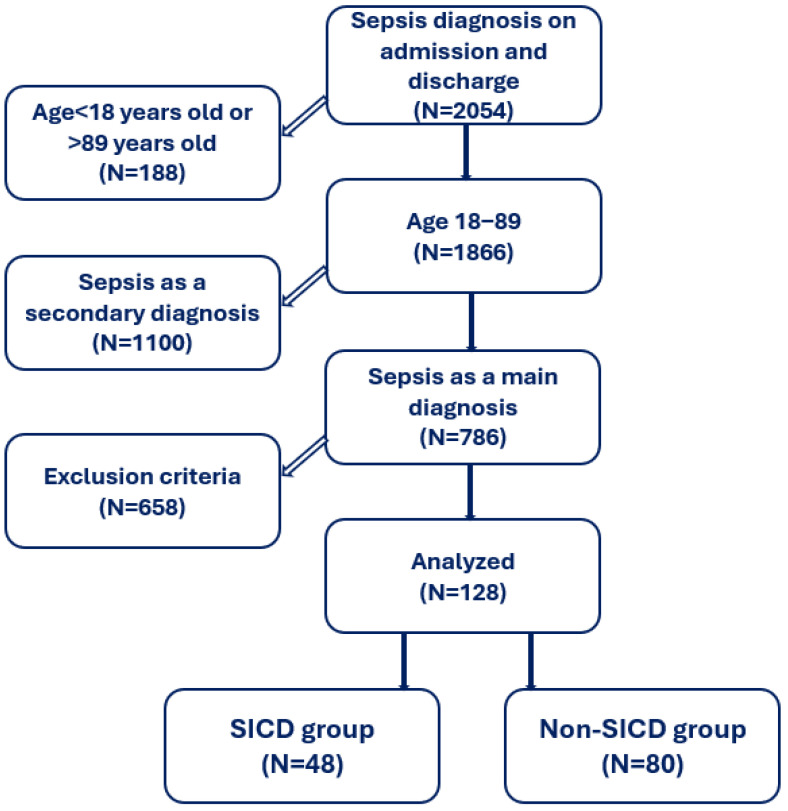
Study flowchart. SICD = Sepsis-induced cardiac dysfunction, Non-SICD = Non-Sepsis-induced cardiac dysfunction.

We conducted a retrospective, observational study on patients admitted and discharged with sepsis or septic shock as the primary diagnosis at a tertiary hospital between February 2023 and October 2024 (Figure 1). We used this system in order to exclude any patients with a false positive diagnosis (sepsis on admission, which was later disconfirmed) and to exclude any patients who developed sepsis over the hospital stay. The ethics committee of Elias University Emergency Hospital approved this study (02022023-2/02.02.2023).

The study enrolled patients admitted and discharged with a diagnosis of sepsis or septic shock, aged 18–89 years old, who required intensive care unit admission or who required monitoring in a special care unit within other departments of the hospital. Only patients who had a transthoracic echocardiography (TTE) performed within 24 h of admission were included. If the TTE showed new onset LV systolic dysfunction, a second TTE was conducted to determine the persistence or resolution of LV systolic dysfunction in survivors. Patients with sepsis or septic shock upon admission and without significant cardiovascular disease were enrolled—inclusion and exclusion criteria are detailed in Table 1.

### 2.2. Definitions

Sepsis and septic shock were defined according to the Sepsis-3 criteria [1]. The sepsis diagnosis was categorised based on the primary site of infection, the source of infection (community-acquired or healthcare-associated), and the type of causative microorganism. If more than one pathological microorganism was identified in different site cultures, the clinical and imaging data were reviewed, and a primary localisation was determined.

The diagnosis of SICD was defined as previously unknown or new-onset LV systolic dysfunction with an LVEF of less than 50%. In patients with pre-existing LV systolic dysfunction, the diagnosis of SICD was established when a reduction of at least 10% from baseline was observed. The reversible nature of cardiac dysfunction could only be assessed in patients who survived at least 7 days of hospital stay. All echocardiographic examinations were acquired and interpreted by experienced cardiologists. LV systolic function was evaluated using the Simpson biplane method whenever feasible. In patients with a poor acoustic window, LVEF was evaluated visually. Right ventricular longitudinal function was assessed using the Tricuspid Annular Plane Systolic Excursion (TAPSE) and s wave. A TAPSE over 17 mm and an s wave of over 10 cm/s were considered normal. LV diastolic function was assessed using E wave, lateral e’ wave, septal e’ wave, average e’ wave, E/lateral e’ ratio, E/septal e’ ratio, and E/average e’ ratio. Isolated LV diastolic dysfunction was considered in patients with preserved LV and RV systolic function and with one of the following: E/lateral e’ > 10, E/septal e’ > 10, or E/average e’ > 10.

### 2.3. Outcomes

The following outcomes were established: in-hospital mortality, prolonged ICU stay (defined as over 7 days of hospitalization in the ICU) and prolonged hospital stay (defined as over 28 days of hospitalization). The primary outcome was a composite of in-hospital mortality, prolonged hospital stay, and prolonged ICU stay.

### 2.4. Statistical Analysis

Statistical analyses were performed using IBM SPSS Statistics for Windows, version 26.0 (IBM Corp., Armonk, NY, USA). Continuous variables were assessed for normality using the Shapiro–Wilk test. Normally distributed data are reported as means with standard deviations, and non-normally distributed data were reported as medians with interquartile ranges. Categorical variables are reported as frequencies and percentages.

Comparisons between groups for continuous variables were conducted using Student’s *t*-test for normally distributed data and the Mann–Whitney U test for non-normally distributed data. Comparisons between more than two independent groups of variables with non-normal distributions were performed using the Kruskal–Wallis test. The chi-square test was performed to compare categorical variables (or Fisher’s exact test, when frequencies were less than 5). A *p*-value of <0.05 was considered statistically significant. To identify risk factors associated with the development of SICD, univariate logistic regression analyses were performed. Odds ratios (OR) with 95% confidence intervals (CI) were reported. A *p*-value < 0.05 was considered statistically significant.

## 3. Results

### 3.1. Baseline Characteristics

A total of 128 patients were included (out of 2054 septic patients screened), the median age was 72.5 years (IQR 63, 81), and 55% were male. More than a third of these patients developed SICD (37%). Baseline characteristics are shown in Table 2. Age and sex did not differ significantly between patients with SICD vs. those without SICD.

All patients underwent TTE within 24 h from admission. Among the 48 patients who developed SICD, 9 died before day 7, so no follow-up TTE could be performed.

The percentage of patients with chronic alcohol consumption was higher in the SICD group than in the non-SICD group. Chronic alcohol consumption was identified as a risk factor for developing isolated LV systolic dysfunction VD (OR 4.4, 95% CI: 1.6–11.7, *p* = 0.003) and biventricular systolic dysfunction (OR 5.4, 95% CI: 1.01–28.9, *p* = 0.048).

SICD patients were more likely to develop septic shock throughout hospital stay than patients without SICD (75% vs. 56%, *p* = 0.033). The need for orotracheal intubation on admission was a risk factor for developing SICD, even after adjusting for age and sex (OR 2.9, 95% CI: 1.2–7.4, *p* = 0.020). Similarly, the need for vasopressor therapy initiation from hospital admission was a risk factor for developing SICD, even after adjusting for age and sex (OR 2.5, 95% CI: 1.2–5.3, *p* = 0.016).

### 3.2. NT-proBNP

In the present study, a total of 90% of patients had NT-proBNP drawn on admission and the majority of those (99%) had elevated values. Patients who developed any type of cardiac dysfunction had higher NT-proBNP values compared to those who did not: 9022 pg/mL vs. 4000 pg/mL, but without reaching statistical significance (Table 2). NT-proBNP over 9000 pg/mL in this group of patients was identified as a predictor of developing SICD of any type: OR 3.12, 95% CI: 1.4–6.8, *p* = 0.004. Furthermore, NT-proBNP over 9000 pg/mL was a significant predictor of the composite outcome, even after adjustment for age and sex (OR 7.7, 95% CI: 3.3, 17.8, *p* < 0.001) and also a predictor of mortality even after adjustment for age and sex (OR 7.8, 95% CI: 3.2, 18.7, *p* < 0.001). These findings highlight the importance of NT-proBNP as a prognostic marker in septic patients, even in the absence of echocardiographic evidence of cardiac dysfunction.

Analyzing NT-proBNP values by each SICD subtype revealed the following results: The highest median value was observed in the isolated LV systolic dysfunction group—9980 pg/mL (IQR 3100, 12,933), followed by the isolated diastolic dysfunction group: 9022 pg/mL (IQR 4180, 13,200). The isolated RV systolic dysfunction group had an NT-proBNP median value of 6107 pg/mL (IQR 2727, 13,000) and the LV biventricular dysfunction group had a median value of 5660 pg/mL (IQR 1430, 9022).

### 3.3. Troponin

A total of 58% of patients had a troponin I level drawn on admission (upper reference limit: 0.120 ng/mL). A proportion of 43% had elevated levels of troponin; most of these had only slightly raised values. Median troponin I levels were significantly higher in non-survivors vs. survivors (0.223 [0.07, 1.1] vs. 0.0 [0.0, 0.2], *p* = 0.02). There was no significant difference in troponin I between patients with SICD and those without SICD. There was no correlation between troponin I levels and any type of SICD identified in this study.

### 3.4. Echocardiography

**Figure 2:**
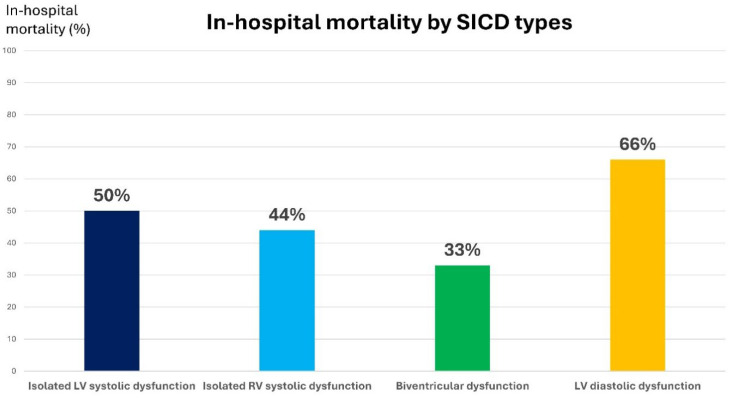
In-hospital mortality by specific Sepsis-induced cardiac dysfunction (SICD) type. LV = left ventricle, RV = right ventricle.

We identified four different types of cardiac dysfunction based on echocardiography (Graphical Abstract, Table 3): isolated LV dysfunction, isolated RV dysfunction, biventricular dysfunction, and isolated LV diastolic dysfunction. Patients with isolated LV systolic dysfunction were significantly younger than the rest of the group (median age 57 vs. 73, *p* = 0.039). There was a male predominance in the isolated LV systolic dysfunction group and the biventricular dysfunction group. Mortality rates did not differ significantly among the SICD groups. The highest mortality rate was observed within the diastolic dysfunction group (Figure 2).

Patients who developed SICD of any kind had significantly lower lateral and septal e’ wave values and considerably higher values of E/lateral e’ ratio, E/septal e’ ratio, and E/average e’ ratio (Table 4).

### 3.5. Outcomes

Patients with SICD had more extended hospital stays and longer ICU stays than patients without cardiac dysfunction. A total of 56 patients (44%) experienced in-hospital mortality. Even though mortality rates did not statistically significantly differ, SICD patients had higher mortality rates vs. those without cardiac dysfunction (54% vs. 37%, *p* = 0.066). Even though the occurrence of SICD did not significantly predict mortality alone in this group, it was a significant predictor of composite outcome (OR = 2.211, 95% CI: 1–4.6, *p* = 0.037), even after adjusting for age and sex (OR 2.2, 95% CI: 1–4.7, *p* = 0.039).

## 4. Discussion

This study aimed to identify patients with SICD, describe their echocardiography patterns and question their outcome. There is currently no universally accepted definition or standardised diagnostic test for this condition. Furthermore, identifying cardiac dysfunction in critically ill patients can be challenging. Critically ill patients admitted to the ICU can have suboptimal acoustic windows due to several factors: invasive or non-invasive mechanical ventilation, patient positioning, or chest wall oedema, which can lead to poor echographic images, difficulties in underlining the endocardial border, and challenges in obtaining angle-dependent measurements (such as e’ waves) [14]. The feasibility of acquiring high-quality echocardiography images in the ICU can be a challenge. As a result, a proportion of patients in our study had a visually assessed LVEF reported or a visually assessed RV function noted.

Even though SICD is generally believed to be a transient decrease in LV systolic function, multiple studies revealed that this definition is not sufficient. We identified four different patterns of cardiac dysfunction: isolated LV systolic dysfunction, biventricular dysfunction, isolated RV dysfunction, and isolated LV diastolic dysfunction. The reported incidence of SICD in the literature varies across studies due to differences in the definitions used and the types of cardiac dysfunction included. The reported incidence of LV systolic dysfunction assessed by LVEF is around 20% and the incidence of RV dysfunction is around 35% [5,15]. When LV function was assessed by global longitudinal strain, the incidence of cardiac dysfunction was higher [16]. Diastolic dysfunction parameters, including E and E/A ratio, are load-dependent [17]. As e’ wave and E/e’ ratio seem to be less load dependent, we chose to report them [18]. Previous reports showed an association between lower e’ and higher E/e’ ratio values and mortality in septic patients [19].

Patients who developed LV systolic dysfunction were significantly younger than the rest of the group, similar to findings reported by Sato et al. [7]. Also, there was a male predominance in the isolated LV systolic and biventricular dysfunction groups. Similarly, other studies reported a male predominance both in sepsis and in SICD [7,8,20,21]. The need for vasopressor therapy and the need for orotracheal intubation on hospital admission were identified as predictors for developing SICD of any kind, which suggests strong prognostic implications. An important observation in the present analysis was that chronic alcohol consumption emerged as a risk factor for developing isolated LV systolic dysfunction and biventricular dysfunction. Previous reports state that patients with chronic alcohol abuse are more at risk to develop sepsis, septic shock and worse outcome [22,23]. To the best of our knowledge, this is the first analysis to suggest an association between chronic alcohol consumption and the risk of SICD in septic patients.

Mortality rates were higher in patients who developed SICD of any type vs. patients who did not develop cardiac dysfunction (54% vs. 37%), but the analysis did not reach statistical significance. However, the *p* value of 0.066 trends towards statistical significance, indicating relevant prognostic value. Furthermore, SICD patients had more extended hospital and ICU stays. Previous studies reported an association between LV systolic dysfunction and worse outcomes and increased mortality [5,6]. The highest in-hospital mortality was reported within the diastolic dysfunction group (66%), which might be partially explained by the advanced age in this group (median age 80 years). Similarly, diastolic dysfunction in septic patients was also reported to have worse outcomes [24,25], and RV dysfunction was associated with increased mortality [26,27].

Even though the occurrence of SICD of any kind did not significantly predict mortality alone in this group, it was a significant predictor of composite outcome, even after adjusting for age and sex. Gathering this information and the previously reported results, it is clear that cardiac dysfunction in sepsis is not a benign, reversible condition, but a predictor of worse outcomes.

NT pro-BNP is secreted by the myocardium as a response to stretch, which could occur in cardiac dysfunction (preexisting or sepsis-related) or as a response to disproportionate fluid administration. According to current guidelines, NT-proBNP is an essential component in establishing the diagnosis of heart failure in patients with suggestive signs and symptoms [28,29]. Guidelines also mention that patients with sepsis might have elevated NT-proBNP levels [28]. In the current analysis, 90% of patients had a NT-proBNP level measurement on hospital admission, and of those, 99% had elevated NT-proBNP values (over 125 ng/mL). Only one patient had a normal natriuretic peptide value. Similarly, Vassali et al. reported elevated NT-proBNP values in 93.4% of the septic patients analysed and Masson et al. reported abnormal values in 97.4% of patients with severe sepsis and septic shock [30,31].

The highest median NT-proBNP values were reported in the isolated LV systolic dysfunction group, similar to previous reports [24].

NT-proBNP levels upon admission of over 9000 pg/mL in this group of patients were a significant predictor of developing SICD of any type. This is an important observation that shows the elevated incidence of high NT-proBNP levels in septic patients. The value identified is much higher than the reported cut-off values for acute heart failure. The ICON-RELOADED Study reported NT-proBNP cut-off values for “rule-in” algorithm for acute heart failure in the Emergency Department: >450 pg/mL in patients younger than 50 years old, >900 pg/mL for patients 50–75 years old and >1800 pg/mL for patients older than 75 years old [32]. The NT-proBNP cut-off value identified in this analysis used in the emergency department could lead to an early identification of patients who are at risk of developing SICD and would impose a closer cardiovascular surveillance.

NT pro-BNP values on admission were significantly higher in non-survivors compared to survivors in this cohort (3172 [2253, 5657] vs. 9210 [7400, 15,809], *p* < 0.001). Furthermore, high NT-proBNP values (over 9000 pg/mL) were identified as a predictor of the composite outcome, even after adjustment for age and sex, and also a predictor of mortality, even after adjustment for age and sex. These findings highlight the importance of NT-proBNP as a prognostic marker in septic patients, even in the absence of echocardiographic evidence of cardiac dysfunction. Although elevated values can also be present in other clinical conditions, such as renal dysfunction and atrial fibrillation, natriuretic peptides are valuable prognostic markers in sepsis. They could aid in early clinical stratification alongside clinical scoring systems (such as SOFA, APACHE-II, SAPS-II) [33].

Myocardial injury, evidenced by elevated troponin levels, is an established phenomenon in sepsis, especially in critically ill patients [34]. Elevated troponin levels are not considered diagnostic criteria of SICD because cardiac dysfunction can develop even in the absence of troponin elevation. Other studies reported troponin as a marker of prognostic value in sepsis [35,36]. In our study, high troponin levels were not associated with SICD. However, troponin had predictive value, as patients who died had significantly higher troponin levels than those who survived.

The study offers important clinical applications. The findings suggest that younger patients and chronic alcohol abusers may be particularly at risk to develop systolic dysfunction during sepsis. Elevated NT-proBNP levels (with an identified cut-off of 9000 pg/mL) were associated with an increased risk of developing SICD and of poor prognosis. Furthermore, this analysis describes the heterogeneity of cardiac dysfunction in sepsis.

Future perspectives in SICD include establishing a universally recognized terminology, definition and a set of diagnostic criteria. A severity score, which would include clinical status upon admission and high NT-proBNP levels might help in early diagnosis of SICD and better monitoring. AI-ECG algorithms validated for septic patients could play an important part in the early detection, and even the prediction, of systolic or diastolic cardiac dysfunction, especially in low resource centers, where echocardiography may not be widely available.

### Study Limitations

The present study has several limitations. Firstly, the generalizability of the results is limited due to the relatively small sample size and retrospective nature of the analysis. Secondly, echocardiography reports were not comprehensive in all patients due to poor acoustic windows in critically ill patients (influenced by patient positioning, ventilation, and comorbidities).

## 5. Conclusions

This study describes the incidence and spectrum of SICD in a group of septic patients admitted to the intensive care unit in a tertiary care hospital. Chronic alcohol consumption emerged as a risk factor for developing isolated LV and biventricular systolic dysfunction. The four types of cardiac dysfunction identified were: isolated LV dysfunction, isolated RV dysfunction, biventricular dysfunction, and isolated LV diastolic dysfunction. Cardiac dysfunction associated with sepsis and elevated NT-proBNP levels had strong prognostic implications. The occurrence of any type of cardiac dysfunction was a significant predictor of composite outcome (prolonged hospital stay, ICU stay and mortality). Elevated NT-proBNP levels (over 9000 pg/mL) were significant predictors of developing any type of SICD, mortality and composite outcome.

## Figures and Tables

**Table 1:** Inclusion and exclusion criteria.

Inclusion	Exclusion
Sepsis or septic shock as the main admission and discharge diagnosis	Cardiovascular: o History of cardiomyopathy with systolic dysfunction (LVEF < 40%) or HFrEF o Preexistent or newly diagnosed severe aortic or mitral valve disease o Infective endocarditis o Acute coronary syndrome o Acute intermediate or high risk PE
Age 18–89 years old	Oncological o Advanced or end stage cancer or hematological disease
TTE in the first 48 h from admission Repeat TTE within 10 days if there is newly diagnosed LV dysfunction on first TTE	Preexistent end stage disease/ organ failure o Hepatic o Neurological o Renal (without any replacement therapy) o Pulmonary

HFrEF—heart failure with reduced ejection fraction, LV—left ventricle, LVEF—left ventricle ejection fraction, PE—pulmonary embolism, TTE—transthoracic echocardiography.

**Table 2:** Baseline characteristics.

	SICD (Total = 48)	Non-SICD (Total = 80)	*p*
Age (years), median [IQR]	74 [66.5, 82]	71.5 [62.5, 80]	0.248
Male sex, number (%)	25 (52)	46 (57)	0.551
Smoker, number (%)	12 (25)	20 (25)	1
Alcohol abuse, number (%)	11 (23)	11 (14)	0.183
Hospital stay (days), median [IQR]	12 [7, 19.5]	10 [7, 18]	0.752
ICU stay (days), median [IQR]	3.5 [1, 7]	2 [1, 7]	0.242
SOFA on admission, median [IQR]	7 [5, 9]	6 [4, 9]	0.470
OTI on admission, number (%)	14 (29)	10 (12)	**0.019**
Vasopressor on admission, number (%)	31 (65)	33 (42)	**0.013**
In-hospital mortality, number (%)	26 (54)	30 (37)	0.066
Septic shock, number (%)	36 (75)	45 (56)	**0.033**
Healthcare-associated infection, number (%)	18 (37)	32 (40)	0.779
Positive cultures, number (%)	37 (77)	64 (80)	0.695
Comorbidities Atrial fibrillation Chronic lung disease (COPD, asthma) Diabetes mellitus type 2 Obesity Chronic hepatitis Chronic kidney disease Neurodegenerative disease History of stroke	19 (40) 8 (17) 13 (27) 6 (12) 4 (8) 8 (17) 25 (52) 10 (21)	35 (44) 12 (15) 32 (40) 10 (12) 5 (6) 17 (21) 35 (44) 24 (30)	0.644 0.801 0.138 1 0.727 0.647 0.360 0.256
Cardiac biomarkers Troponin I (ng/mL), median [IQR] NT-proBNP (pg/mL), median [IQR]	0.16 [0.0, 0.71] 9022 [2465, 13,724]	0.06 [0.0, 0.27] 4000 [3000, 10,100]	0.368 0.172

COPD = Chronic obstructive pulmonary disease, ICU = intensive care unit, IQR = interquartile range, OTI = orotracheal intubation, SOFA = Sequential Organ Failure Assessment, SICD = sepsis-induced cardiac dysfunction.

**Table 3:** Types of cardiac dysfunction.

	SICD: Isolated LV Systolic Dysfunction	SICD: Isolated RV Systolic Dysfunction	SICD: Biventricular Systolic Dysfunction	SICD: Isolated LV Diastolic Dysfunction	Non-SICD	*p* Value
Definition	New and reversible drop in LVEF of at least 10% from baseline and under 50%	New and reversible drop in TAPSE of at least 2 mm from baseline and under 17 mm	LV and RV systolic dysfunction	New and reversible at least one of the following: E/septal e’ > 10 E/lateral e’ > 10 E/average e’ > 10	Without cardiac dysfunction attributable to sepsis	
Number (%) (total = 128 patients)	12 (9)	9 (7)	6 (5)	21 (16)	80 (63)	
Age, years, median (IQR)	57 (49, 76)	73 (67, 82)	73.5 (65, 82)	80 (73, 84)	71.5 (62.5, 80)	**0.038**
Male (number/total)	7/12	2/9	6/6	10/21	46/80	**0.049**
In-hospital mortality (number/total)	6/12	4/9	2/6	14/21	30/80	0.184

IQR = interquartile range, LVEF = left ventricular ejection fraction, VTI LVOT = Velocity time integral left ventricular outflow tract, SICD = sepsis-induced cardiac dysfunction, TAPSE = tricuspid annular plane systolic excursion.

**Table 4:** Echocardiography characteristics.

	SICD	Non-SICD	*p* Value
LVEF, median [IQR], %	50 [41, 63]	55 [53, 60]	0.182
VTI LVOT, median [IQR], cm	17.2 [14.1, 21]	19.5 [17.1, 22.5]	0.070
TAPSE, median [IQR], mm	18 [16, 21]	21 [18, 22]	**0.002**
RV s wave, median [IQR], cm/s	11 [9.6, 12]	12.7 [11.5, 14]	**0.002**
E, median, cm/s	80 [60, 88]	70 [52, 85]	0.409
E/A, median [IQR]	1 [0.7, 1.5]	0.7 [0.6, 1]	0.081
Lateral e’ wave, median [IQR], cm/s	7 [5.6, 9.7]	10.8 [8, 12.3]	**0.003**
Septal e’ wave, median [IQR], cm/s	5 [4, 6.5]	7 [5.1, 9.2]	**0.001**
E/lateral e’, median [IQR]	10 [7.3, 12.9]	6.3 [4.8, 8.5]	**<0.001**
E/septal e’, median [IQR]	12.8 [10.8, 17.1]	9.3 [7.4, 10.9]	**<0.001**
E/average e’, median [IQR]	11.6 [8.6, 15]	7.8 [5.9, 9.4]	**<0.001**

## Data Availability

The data presented in this study are available on request from the corresponding author.
